# Society of Apothecaries

**Published:** 1835-07-01

**Authors:** 


### INTELLIGENCE.

### SOCIETY OF APOTHECARIES.

*Regulations to be observed by Students intending to qualify themselves to practise as Apothecaries in England and Wales.*

The Court of Examiners of the Society of Apothecaries of London have witnessed, with great satisfaction, the benefits derived from the course of study enjoined by them, in the increased acquirements of the candidates who present themselves for examination; and, being assured that the time is arrived when it behoves them to complete the scheme which they have long had in view, and to which they have advanced by successive and cautious steps, they now publish an extended course of study, which, although it may perhaps require hereafter some modification in the details, may be considered, both in extent and duration, as final.

In prolonging the period of study, the Court feel confident that they are consulting the interests of the public, and that they are also acting in accordance with the wishes of the profession generally, and more especially of the enlightened body of gentlemen engaged in teaching medicine, and the various sciences connected therewith, who have, for some time past, expressed their sense of the great advantages which would result from a systematic arrangement of the sessions at the medical schools, and of the particular subjects of study appropriate for the winter and summer seasons. The Court will be solicitous to lessen whatever inconvenience may, in the first instance, be attendant upon this important change; and they will be ready to pay attention to the cases of such students as may be prevented, by peculiar circumstances, from commencing their attendance at the schools in the early part of October; the period of the year at which it is most especially desirable that such attendance should, in future, commence.

The liberality of the physicians of the London hospitals, in promptly acceding to the wishes of the Court, that students might have afforded to them a more extended opportunity of studying practical medicine, without any augmentation of expense, has enabled the Court to require an attendance of the student for eighteen months at an hospital, instead of twelve; and to this boon the physicians would add a yet more essential service, by inducing the governors of the hospitals with which they are connected, to reorganise their respective out-patient establishments, and afford to students an opportunity of studying large and important classes of disease, which are very rarely admitted within the wards of an hospital.

The great advantages which students have derived from a regular course of periodic examinations, in the schools in which this system has been adopted, associated with a systematic and combined course of reading and oral instruction, induce the Court again to press this subject especially upon the attention of teachers.

The use of a class-book, also, for each particular branch of study, would better enable the student to reduce into order the numerous facts placed before him, and to refer again and again to such points as require a sustained exercise of the powers of reasoning, for their full and clear comprehension.

The Legislature having made an apprenticeship of five years imperative upon all students, and having permitted them to present themselves for examination at the age of twenty-one, obviously intended that the greater part of their medical education should be included within that period; and the court have great pleasure in stating, that in very many instances students have actually completed their course of study, and have been admitted to an examination, with a few weeks after the termination of their apprenticeship. It is, however, to be regretted that this advantage has frequently been lost sight of, and that a great proportion of this valuable time, and not unfrequently the whole term of it, has been passed exclusively in practical pharmacy. The court are desirous of impressing upon parents the necessity of preventing this waste of time, by making such arrangements with practitioners with whom they place their sons, as may enable the young men to commence their attendance upon lectures in the course of the third year of their apprenticeship.

The court renew their recommendation that the apprenticeship should not begin until the youth has attained his seventeenth year, and that he should previously have received a sound classical education, and have been instructed in the elements of mathematics and natural philosophy, and have acquired a knowledge of the French, and, if possible, the German languages.

The period of apprenticeship is by no means to be considered as of small importance; during that time it is incumbent upon the master to take care that his apprenticeship keeps up and extends, by a regular course of reading, both his classical and general knowledge; it is also his duty to ascertain, by occasional examinations, that his pupil is acquiring the elements of professional knowledge; and that he becomes acquainted with the nomenclature of the profession, the manipulations of pharmacy, and the elements of osteology; whilst opportunities should be afforded him of watching the progress of disease, and of noticing the effects of remedies.

The court have reason to believe, that students would in many instances gladly avail themselves of an opportunity of passing their Latin examination upon the commencement of their studies at the medical schools; the court have, therefore, arranged a plan for that purpose, which may be adopted at the option of the student, at the time of registering his first attendance upon lectures. After this preliminary examination in Latin has been satisfactorily passed, the student will not be subjected to any farther examination in Latin medical classics.

The Court of Examiners have only to add, that they have framed the following course of study with especial reference to the surgical as well as medical duties which devolve upon the general practitioner when engaged in practice, and with the knowledge that students, with few exceptions, pass an examination in surgery at the Royal College of Surgeons, as well as one in medicine at the Hall; the court have, therefore, taken care to afford every facility for a strict conformity with the regulations of the College, as well as with those which they have themselves enjoined. The court exhort students not to rest satisfied with a mere formal compliance with the injunctions of authority, but to be actuated by still higher motives, and to find in these an incentive to a zealous and generous devotion of their time, their labour, and their best faculties, to the acquisition of an accurate and comprehensive knowledge of the principles of the healing art.

Every candidate for a certificate to practise as an apothecary, will be required to produce testimonials—

* Of having served an apprenticeship of not less than five years to an apothecary;

† Of having attained the full age of twenty-one years;

‡ And of good moral conduct.

Students whose attendance on lectures shall commence on or after the 1st of October, 1835, will also be required to produce proof of having attended, during three Winter and two Summer sessions, lectures in the following order, and medical practice from the commencement of the second, to the termination of the third Winter session.

The Winter medical session is to be understood as commencing on the 1st of October, and terminating in the middle of April, with a recess of fourteen days at Christmas: the Summer session as commencing on the 1st of May, and ending on the 31st of July.

*First Winter Session.*

Chemistry.
Anatomy and physiology.
Anatomical demonstrations.
Materia medica and therapeutics.

*First Summer Session.*

Botany; and such other branches of study as may improve the student's general education.

*Second Winter Session.*

Anatomy and physiology.
Anatomical demonstrations.
Dissections.
Principles and practice of medicine.
Medical practice of an hospital.

* No gentleman practising as an apothecary in England or Wales can give his apprentice a legal title to examination, unless he is himself legally qualified to practise as an apothecary, either by having been in practice prior to or on the 1st of August, 1815, or by having received a certificate of his qualification from the Court of Examiners. An apprenticeship for not less than five years to surgeons practising as apothecaries in Scotland and Ireland, gives to the apprentice a title to be admitted to examination.

† As evidence of age, a copy of the baptismal register will be required in every case where it can possibly be procured.

‡ A testimonial of moral character from the gentleman to whom the candidate has been an apprentice, will always be more satisfactory than from any other person.

*Second Summer Session.*

Botany, if not attended during the first Summer session.
Midwifery and diseases of women and children.
Forensic medicine.
Medical practice of an hospital.

*Third Winter Session.*

Dissections.
Principles and practice of medicine.
Midwifery, with attendance on cases.
Medical practice of an hospital or dispensary.

The student is required to attend the medical practice of a recognised hospital, from the commencement of the second Winter to the termination of the second Summer session, and from that time to the end of the third Winter session, at an hospital, or recognised dispensary.

The sessional course of instruction in each respective subject of study, is to consist of not less than the following number of lectures, viz.:—

One hundred on chemistry.
One hundred on materia medica and therapeutics.
One hundred on the principles and practice of medicine.
Sixty on midwifery, and the diseases of women and children.
Fifty on forensic medicine.
Fifty on botany.

The number of lectures on anatomy and physiology, and of anatomical demonstrations, must be in conformity with the regulations of the Royal College of Surgeons of London, on these subjects.

The lectures required in each course respectively, must be given on separate days.

Students, when they present themselves for examination, must bring testimonials of having received instruction in practical chemistry during their attendance upon the lectures on chemistry, materia medica, or forensic medicine; and also of having attended a full course of clinical lectures, and such instruction in morbid anatomy as may be afforded them during their attendance at an hospital.

Every student will be required to produce proof of having dissected the whole of the body once at least.

Students whose attendance on lectures commenced prior to the 1st of February, 1828, will be admitted to examination in conformity with the regulations published in September, 1826; viz. after an attendance on

One course of lectures on chemistry.
One course of lectures on materia medica.
Two courses of lectures on anatomy and physiology.
Two courses of lectures on the theory and practice of medicine.
And six months physician's practice at an hospital, or nine mouth's at a dispensary.

Those who began to attend lectures subsequently to the 1st of February, 1828, and previously to the 1st of October of the same year, in conformity with the regulations of September, 1827; viz. after an attendance on

One course of lectures on chemistry.
One course of lectures on materia medica and botany.
Two courses of lectures on anatomy and physiology.
Two courses of lectures on the theory and practice of medicine: these last having been attended subsequently to the lectures on chemistry and materia medica, and to one course at least of anatomy.
And six months', at least, physician's practice at an hospital, or nine months' at a dispensary; such attendance having commenced subsequently to the termination of the first course of lectures on the principles and practice of medicine.

Those whose attendance on lectures commenced in October, 1828, must have complied with the regulations of September, 1828; viz. by having attended

Two courses of lectures on chemistry.

Two courses of lectures on materia medica and botany.

Two courses of lectures on anatomy and physiology.

Two courses of anatomical demonstrations.

Two courses of lectures on the theory and practice of medicine; these last having been attended subsequently to one course of lectures on chemistry, materia medica, and anatomy.

And six months, at least, the physician's practice at an hospital, (containing not less than sixty beds), or nine months at a dispensary; such attendance to have commenced subsequently to the termination of the first course of lectures on the principles and practice of medicine.

All students who began to attend lectures in January, 1829, are required to have attended the physician's practice at an hospital for nine months, or at a dispensary for twelve months, and also to have attended

Two courses of lectures on midwifery, and the diseases of women and children.

Students whose attendance on lectures commenced on or after January, 1831, must adduce proof of having devoted at least two years to an attendance on lectures and hospital practice; and of having attended the following courses of lectures:

Chemistry: Two courses—each course consisting of not less than forty-five lectures.

Materia medica and therapeutics: Two courses—each course consisting of not less than forty-five lectures.

Anatomy and physiology: Two courses.

Anatomical demonstrations: Two courses.

Of the same extent as required by the Royal College of Surgeons of London.

Principles and practice of medicine; Two courses—each course consisting of not less than forty-five lectures.—To be attended subsequently to the termination of the first course of lectures on chemistry, materia medica, and anatomy and physiology.

Botany: One course consisting of not less than thirty lectures.—To be attended between the 1st of April and 31st of October.

Midwifery, and the diseases of women and children: Two courses.

Forensic medicine: One course—To be attended during the second year.

Students are likewise earnestly recommended to avail themselves of instruction in morbid anatomy.

The candidate must also have attended, for twelve months at least, the physician's practice at an hospital containing not less than sixty beds, and where a course of clinical lectures is given; or for fifteen months at an hospital wherein clinical lectures are not given; or for fifteen months at a dispensary connected with some medical school recognised by the Court. No part of this attendance can be entered upon until the termination of one entire year from the commencement of attendance on lectures, nor until one course of lectures, at least, on chemistry, materia medica, anatomy, and the practice of medicine, have been attended in the order prescribed by the Regulations.

The testimonials of attendance on lectures, and medical practice, must be given on a printed form, with which students, will be supplied, on application, at the under-mentioned places:

In London, at the beadle's office, at this Hall.

In Edinburgh, at Messrs. Mac Lachlan and Stewart's, booksellers.

In Dublin, at Messrs. Hodges and Smith's, booksellers.

In the provincial towns, where there are medical schools, from the gentlemen who keep the registers of the schools.

No other form of testimonial will be received; and no attendance on lectures will qualify a candidate for examination, unless the lecturer is recognised by the Court.

The names of the lecturers recognised by the Court may be seen on application to the several gentlemen acting as registrars in the provincial schools, and at the beadle's office at the Hall.

The teachers in London, Dublin, Edinburgh, Glasgow, and Aberdeen, recognised by the constituted medical authorities in those places respectively, are recognised by the Court; and certificates given by the medical professors in the continental universities are also recognised and received by the Court.

Gentlemen wishing to be recognised as lecturers, are referred to the following resolutions of the Court, passed on the 18th of November, 1830, viz.

Resolved,—That no member of the Court of Examiners shall be recognised as a lecturer on any branch of medical science.

That the Court will not recognise any teacher who may give lectures on more than two branches of medical science.

That the Court will not recognise a teacher until he has given a public course of lectures on the subject he purposes to teach; but if, after such preliminary courses of lectures, the teacher should be recognised, the student's certificate of attendance on that course will be received.

That the Court will not recognise a teacher until he has produced very satisfactory testimonials of his attainments in the science he purposes to teach, and also of his ability as a teacher thereof, from persons of acknowledged talents and of distinguished acquirements in the particular branch of science in question.

That satisfactory assurance shall also be given that the teacher is in possession of the means requisite for the full illustration of his lectures, viz. that he has, if lecturing—

On chemistry, a laboratory and competent apparatus;

On materia medica, a museum sufficiently extensive:

On anatomy and physiology, a museum sufficiently well furnished with preparations, and the means of procuring recent subjects for demonstration;

On botany, a hortus siccus, plates or drawings, and the means of procuring fresh specimens;

On midwifery, a museum, and such an appointment in a public midwifery institution as may enable him to give his pupils practical instructions.

That the lecturer on the principles and practice of medicine must be, if he lectures in London, or within seven miles thereof, a Fellow, Candidate, or Licentiate of the Royal College of Physicians of London; and if he lectures beyond seven miles from London, and should not be thus qualified, he must be a graduated Doctor of Medicine of a British university of four years' standing (unless previously to his graduation he had been for four years a Licentiate of this Court).

That the lecturer on materia medica and therapeutics must be a Fellow, Candidate, or Licentiate, of the Royal College of Physicians of London; a graduated Doctor of Medicine of a British University of four years' standing (unless previously to his graduation he had been for the same length of time a Licentiate of this Court); or he must be a Licentiate of this Court of four years standing.

That the lecturer on anatomy and physiology must either be recognised by the Royal College of Surgeons of London, or must be a member of that College of four years' standing.

That the Demonstrator of Anatomy must either be recognised by the Royal College of Surgeons of London, or must be a member of that College.

*Hospitals as Schools of Practical Medicine.*

No hospital (not already recognised) will in future be placed upon the list of recognised schools of practical medicine, unless it is situated in London, or in one of the provincial cities or towns in which schools of medicine are established, and the physicians attached to it give a full course of instruction in clinical medicine and morbid anatomy.

The hospital must contain one hundred patients at least, and must be under the care of at least two physicians, each of whom must be a Fellow, Candidate, or Licentiate of the Royal College of Physicians of London, if the hospital be ituated in London; and if in a provincial town, the physicians, if not members of the Royal College of Physicians, must be graduated Doctors of Medicine of a British university.

The apothecary of the hospital must be legally qualified, either by having been in practice prior to or on the 1st of August, 1815, or by having received a certificate of qualification from the Court of Examiners.

*Dispensaries as Schools of Practical Medicine.*

The court will recognise, as schools of practical medicine, such dispensaries as shall give satisfactory evidence on the following points, viz.

That the Dispensary is situated in some city or town in which there is a medical school recognised by the Court;

That the rules for the government of the Dispensary permit the attendance of students, and that the physicians afford them instruction and opportunities of acquiring practical knowledge in medicine;

That the Dispensary (if within the limit of the jurisdiction of the Royal College of Physicians of London) is under the medical care of at least two physicians, each of whom is a Fellow, Candidate, or Licentiate of the Royal College; and if beyond these limits, that it is under the care of at least two physicians, who, if not so qualified, are graduated doctors of medicine of a British University, of four years' standing.

And that the apothecary of the Dispensary is legally qualified, either by having been in practice prior to or on the 1st of August, 1815, or by having received a certificate of qualification from the Court of Examiners.

*Registration.*

A book is kept at the hall of the Society for the registration, at stated times, of the names of students, and of the lectures, hospitals, or dispensaries, they attend.

All students, in London, are required to appear personally, and to register the several classes for which they have taken tickets; and those only will be considered to have complied with the regulations of the Court whose names and classes in the register correspond with the testimonials of the teachers.

The book will be open for the registration of tickets authorising the attendance of students on lectures and medical practice during the first twenty-one days of October, and the first fourteen days of May, from nine o'clock until two; and for the registration of certificates of having duly attended such lectures or medical practice during the last fourteen days of April and of July.

The Court also require students at the provincial medical schools to register their names in their own hand-writing, in the order above stated, with the registrar of each respective school; and the registrars are requested to furnish the Court of Examiners with a copy of each registration immediately after its termination, as those students only will be admitted to examination whose registrations have been duly communicated to the Court.

*Names of Gentlemen having the Care of the Registers.*

*Bath.*—R. T. Gower, Esq., Lecturer on Anatomy; John Spender, Esq. ditto.
*Birmingham.*—W. Sands Cox, Esq. Lecturer on Anatomy.
*Bristol.*—Dr. Wallis, Lecturer on Anatomy; Henry Clark, Esq. ditto.
*Hull.*—Edward Wallis, Esq. Lecturer on Anatomy; Robert Craven, Esq. ditto.
*Leeds.*—Thomas Pridgen Teale, Esq. Lecturer on Anatomy.
*Liverpool.*—William Gill, Esq. Lecturer on Anatomy.
*Manchester.*—Joseph Jordan, Esq. Lecturer on Anatomy; Thomas Turner, Esq. ditto; Thomas Fawdington, Esq. ditto.
*Sheffield.*—Wilson Overend, Esq. Lecturer on Anatomy; W. Jackson, Esq. ditto.

Each student, at his first registration, will receive the printed form on which he is to obtain the printed certificates of his teachers.

*Examination.*

Every person offering himself for examination must give notice in writing to the clerk of the society, on or before the Monday previously to the day of examination, and must also, at the same time, deposit all the required testimonials at the office of the beadle; where attendance is given every day (except Sunday), from nine until two o'clock.

The examination of the candidate for a certificate of qualification to practise as an apothecary, will be as follows:—

In translating parts of Celsus de Medicina, and Gregory's Conspectus Medicinæ Theoreticæ:*

In physicians' prescriptions, and the Pharmacopœia Londinensis;

In chemistry;

In materia medica and therapeutics;

In botany;

In anatomy and physiology;

In the principles and practice of medicine.†

The examination of a candidate for a certificate of qualification to act as an assistant to an apothecary, in compounding and dispensing medicines, will be as follows:—

In translating physicians' prescriptions, and parts of the Pharmacopœia Londinensis;

In pharmacy and materia medica.

By the 22d section of the act of parliament, no rejected candidate for a certificate to practise as an apothecary, can be re-examined until the expiration of six months from his former examination; and no rejected candidate as an assistant until the expiration of three months.

The Court meet in the Hall every Thursday, where candidates are required to attend at a quarter before four o'clock.

The Act directs the following sums to be paid for certificates;—

For London, and within ten miles thereof, ten guineas.

For all other parts of England and Wales, six guineas.

Persons having paid the latter sum become entitled to practise in London, and within ten miles thereof, by paying four guineas in addition.

For an assistant's certificate, two guineas.

By order of the Court, JOHN WATSON, *Sec.*

*Apothecaries' Hall; April* 23, 1835.

For information relative to these regulations, students are referred to Mr. Watson, who may be seen at his residence, 43, Berners-Street, between the hours of nine and ten o'clock every morning (Sunday excepted); and for information on all other subjects connected with the "Act for better regulating the Practice of Apothecaries," application is to be made to Mr. R. B. Upton, Clerk of the Society, who attends at the Hall every day (Sunday excepted) from one to three o'clock.

It is expressly ordered by the Court of Examiners, that no gratuity be received by any officer of the Court.

* Students may undergo their Latin examination in these works at the commencement of their studies in London, by giving notice to the beadle, at their first registration, of their wish to do so. And students who are already registered will be admitted to this examination on making an application to the Court.

† This branch of the examination embraces an inquiry into the diseases of pregnant and puerperal women; and also into the diseases of children.

